# The outcomes of endoscopic ultrasound‐guided tissue acquisition for small focal liver lesions measuring ≤2 cm

**DOI:** 10.1002/deo2.70031

**Published:** 2024-10-21

**Authors:** Yuichi Takano, Naoki Tamai, Masataka Yamawaki, Jun Noda, Tetsushi Azami, Fumitaka Niiya, Fumiya Nishimoto, Naotaka Maruoka, Tatsuya Yamagami, Masatsugu Nagahama

**Affiliations:** ^1^ Department of Internal Medicine Division of Gastroenterology, Showa University Fujigaoka Hospital Kanagawa Japan

**Keywords:** endoscopic ultrasound, focal liver lesion, intrahepatic cholangiocarcinoma, metastatic liver tumor, tissue acquisition

## Abstract

**Objectives:**

Endoscopic ultrasound‐guided tissue acquisition (EUS‐TA) for focal liver lesions has gained attention as an alternative to percutaneous biopsy. Although the outcomes of EUS‐TA for focal liver lesions have been reported to be favorable, no studies have focused on small focal liver lesions (≤2 cm). The aim of this study was to evaluate the outcomes of EUS‐TA for small focal liver lesions (≤2 cm).

**Methods:**

The details of EUS‐TA performed for focal liver lesions between 2016 and 2022 were retrospectively reviewed. The outcomes were compared between cases involving ≤2 cm lesions and those involving >2 cm lesions. The primary outcomes were diagnostic ability and adverse events.

**Results:**

EUS‐TA for focal liver lesions was performed in 109 cases. Of the 109 cases, 32 (29.3%) involved ≤2 cm lesions and 77 (70.6%) involved >2 cm lesions. Right lobe lesions and transduodenal puncture were significantly fewer in the ≤2 cm group. There were no significant differences in needle gauge, needle type, or number of punctures between the groups. The sensitivity, specificity, and accuracy rates were 96.8%, 100%, and 96.8%, respectively, in the ≤2 cm group and 97.4%, 100%, and 97.4%, respectively, in the >2 cm group, with no significant differences between the groups. There was no difference in adverse events between the groups (0% in the ≤2 cm group and 2.3% in the >2 cm group).

**Conclusions:**

EUS‐TA for small focal liver lesions measuring ≤2 cm has favorable outcomes, which are similar to those for lesions measuring >2 cm.

## INTRODUCTION

Endoscopic ultrasound‐guided tissue acquisition (EUS‐TA), which was first reported by Vilmann et al. in 1992,^1^ has become an essential diagnostic modality in the current clinical practice. Most studies regarding EUS‐TA have focused on solid pancreatic lesions and have reported that it has high diagnostic ability and safety.^2^ Conversely, its accuracy rate has been reported to decrease in cases involving small solid pancreatic lesions (≤2 cm).^3^

In recent years, EUS‐TA has gained attention as an alternative to percutaneous biopsy for focal liver lesions.^4^ Although the outcomes of EUS‐TA for focal liver lesions have been reported to be favorable, no studies have focused on the outcomes for small focal liver lesions (≤2 cm). The aim of this study was to compare the outcomes of EUS‐TA for focal liver lesions 2cm or less and larger than 2cm.

## METHODS

This is a single‐center, retrospective, observational study that was approved by the Ethics Committee of Showa University (Approval number: 2023‐195‐B). The study was conducted in accordance with the tenets of the Declaration of Helsinki.

The study included patients who underwent EUS‐TA for local liver lesions between 2016 and 2022. Cases in which needle puncture was not possible owing to intervening blood vessels or other organs (such as the gallbladder) were excluded. The clinical backgrounds and details of EUS‐TA were retrospectively reviewed using the medical records. The primary outcomes were the diagnostic ability and adverse events of EUS‐TA. The outcomes were compared between cases involving lesions ≤2 cm in size and those involving lesions >2 cm in size.

### Definition

Tumor size was defined as the largest diameter measured by EUS. Cases histologically diagnosed as malignant tumors (carcinoma, adenocarcinoma, gastrointestinal stromal tumor, malignant lymphoma, angiosarcoma, and neuroendocrine neoplasm) were considered malignant. Furthermore, cases diagnosed as class IV or higher by cytological examination were defined as malignant. Cases that were histologically diagnosed as benign but subsequently diagnosed as malignant (e.g., tumor growth on imaging with elevated tumor marker levels) were also defined as malignant. Histologically confirmed benign results with no tumor growth after a 1‐year follow‐up indicated a benign state.

The primary outcomes were diagnostic ability and adverse events. The diagnostic ability of EUS‐TA was defined as the proportion of diagnoses obtained by EUS‐TA that were identical to the final diagnosis. The definition of adverse events established by the workshop of the American Society of Gastrointestinal Endoscopy was used.^5^

### Our institutional strategy for focal liver lesions

In principle, the first choice at our facility for focal liver lesions is EUS‐TA. In cases wherein percutaneous biopsy is technically easy (large lesions of >5 cm in size or lesions on the liver surface), percutaneous biopsy may be selected at the discretion of the operator. In cases wherein EUS‐TA is difficult (surgically altered anatomy, pharyngeal or esophageal stricture, and cardiorespiratory failure wherein sedation is difficult), percutaneous biopsy is also selected.

### EUS‐TA procedure

**FIGURE 1:**
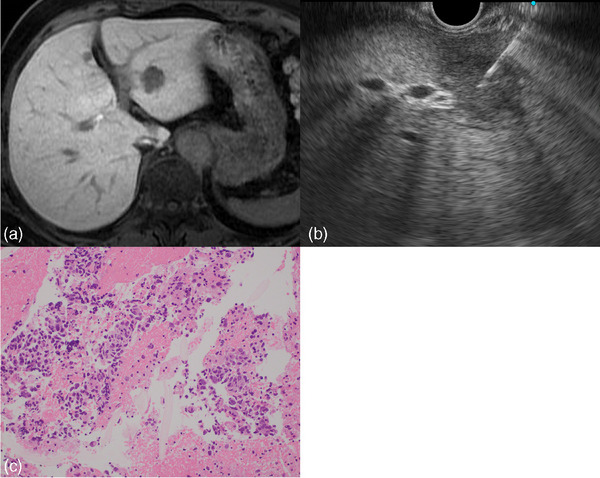
(a) Contrast‐enhanced magnetic resonance imaging revealed a focal liver lesion in segment 2 of the liver. (b) Endoscopic ultrasound showed the largest diameter of the target lesion was 16 mm. Endoscopic ultrasound‐guided tissue acquisition was performed using a 22G needle. (c) Pathologically, the case was diagnosed with liver metastasis from breast cancer (hematoxylin and eosin staining, ×40).

During EUS‐TA, analgesics and sedatives (pethidine hydrochloride [35 mg] or pentazocine [7.5–15 mg] + midazolam [1.0–5.0 mg]) were administered. Furthermore, a GF‐UCT260 endoscope (Olympus Medical Systems, Tokyo, Japan) and a UE‐ME1 or UE‐ME2 observation device (Olympus Medical Systems) were used. The gauge of the puncture needle was 19–25G at the operator's discretion. The number of strokes was 10–20, and the suction pressure was 10–20 mL (negative pressure). If the obtained specimen contained a lot of blood, the slow pull technique was used. Rapid on‐site cytology (ROSE) was not performed. An endoscopist performed macroscopic on‐site evaluation on the obtained specimen, and the procedure was terminated after confirming that a white core tissue had been obtained in principle. In cases with antithrombotic drugs, EUS‐TA was performed in accordance with the Japanese Gastrointestinal Endoscopy Society guidelines (Figure 1).^6^

The needles used were Expect SlimLine (Boston Scientific Japan), Acquire (Boston Scientific Japan), and SonoTip TopGain (Medico's Hirata). Expect SlimLine was a fine‐needle aspiration (FNA) needle, while Acquire and SonoTip TopGain were fine‐needle biopsy needles. Contrast‐enhanced EUS was not performed in this study. The operators were endosonographers with experience in performing EUS‐TA in more than 30 cases.

### Pathological examination

Tissues obtained from EUS‐TA were fixed in formalin, followed by histological diagnosis using hematoxylin and eosin staining. Immunohistochemistry was performed as necessary. After the tissue was fixed in formalin, the remaining liquid component was cytologically examined with Papanicolaou staining. The cytological examination was used as an auxiliary diagnostic approach.

### Statistical analysis

Continuous variables are expressed as medians. Incidence and concordance were compared between the two groups using Fisher's exact test and the Mann–Whitney *U*test as appropriate. Values of *p* < 0.05 were considered to indicate statistical significance.

## RESULTS

### Case selection

**FIGURE 2:**
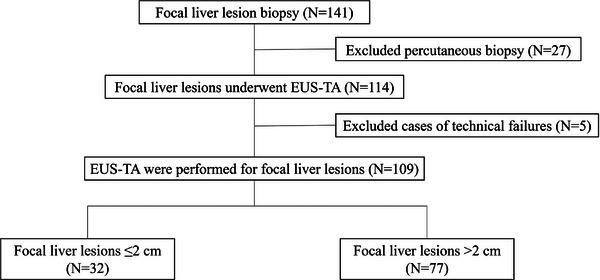
Flowchart of the study subjects.

During the observation period, 141 cases of focal liver lesion biopsy were performed, 27 of which wherein percutaneous biopsy was performed were excluded. EUS‐TA was attempted for focal liver lesions in 114 cases. Of these 114 cases, five were excluded as puncture was not possible (two cases had the gallbladder in the puncture line and two cases had an intervening blood vessel). Ultimately, EUS‐TA was performed in 109 cases. Of these 109 cases, 32 (29.3%) involved a lesion ≤2 cm in size and 77 (70.6%) involved a lesion >2 cm in size. The outcomes of these two groups were compared (Figure 2).

### Clinical background and EUS‐TA procedure

There were no significant differences in age and sex between the two size groups. Right lobe lesions were significantly fewer in the ≤2 cm group (Table 1).

**TABLE 1:** Clinical backgrounds of the two groups.

	Focal liver lesions ≤2 cm (*n* = 32)	Focal liver lesions >2 cm (*n* = 77)	*p‐*value
Age, median (range), years	72 (44–82)	71 (25–90)	0.35
Female, *n* (%)	13 (40.6)	30 (38.9)	0.87
Lesion size, median (range), mm	15 (6–20)	37 (21–170)	‐
Left lobe lesions, *n* (%)	26 (81.2)	49 (63.6)	0.07
Right lobe lesions, *n* (%)	4 (12.5)	24 (31.1)	0.04
Caudate lobe lesions, *n* (%)	2 (6.2)	4 (5.1)	0.36

Table 2 shows a comparison of EUS‐TA procedures and final diagnosis between the two groups. Transduodenal puncture was significantly fewer in the ≤2 cm group. There were no significant differences in needle gauge, needle type, or number of punctures between the groups. Regarding the final diagnosis, metastatic liver tumors were significantly more common in the ≤2 cm group, while intrahepatic cholangiocarcinoma and gallbladder cancer were more common in the >2 cm group.

**TABLE 2:** Details of endoscopic ultrasound‐guided tissue acquisition for focal liver lesions.

	Focal liver lesions ≤2 cm (*n* = 32)	Focal liver lesions >2 cm (*n* = 77)	*p‐*value
Transduodenal puncture, *n* (%)	4 (12.5)	25 (32.4)	0.03
25 gauge needle, *n* (%)	16 (50)	35 (45.4)	0.66
22 gauge needle, *n* (%)	16 (50)	40 (51.9)	0.85
19 gauge needle, *n* (%)	0 (0)	2 (2.5)	0.35
Fine‐needle biopsy needle, *n* (%)	7 (21.8)	25 (32.4)	0.38
Number of punctures, median (range)	2 (1–2)	2 (1–3)	0.49
Immunohistochemistry, *n* (%)	17 (53.1)	48 (62.3)	0.41
Final diagnosis			
Metastatic liver tumor, *n* (%)	24 (75)	24 (31.1)	0.0003
Primary lesion of metastatic tumor			
Colorectal cancer, no. (%)	6 (18.7)	5 (6.4)	0.31
Pancreatic cancer, *n* (%)	5 (15.6)	10 (12.9)	0.72
Breast cancer, *n* (%)	3 (9.3)	4 (5.1)	0.42
Lung cancer, n (%)	3 (9.3)	2 (2.5)	0.16
Cholangiocarcinoma, *n* (%)	1 (3.1)	0 (0)	0.12
Gastrointestinal stromal tumor, *n* (%)	1 (3.1)	1 (1.2)	0.51
Ampulla of Vater cancer, *n* (%)	2 (6.2)	1 (1.2)	0.15
Gallbladder cancer, *n* (%)	1 (3.1)	0 (0)	0.12
Sarcoma of the thigh, n (%)	1 (3.1)	0 (0)	0.12
Pancreatic neuroendocrine neoplasm, *n* (%)	1 (3.1)	1 (1.2)	0.51
Intrahepatic cholangiocellular carcinoma, *n* (%)	2 (6.2)	22 (28.5)	0.01
Hepatocellular carcinoma, *n* (%)	1 (3.1)	12 (15.5)	0.06
Gallbladder cancer, *n* (%)	0 (0)	9 (11.6)	0.04
Malignant lymphoma, *n* (%)	1 (3.1)	4 (5.1)	0.63
Other malignant neoplasms, *n* (%)	0 (0)	4 (5.1)	0.19
Benign lesions, *n* (%)	3 (9.3)	3 (3.8)	0.25

### Outcomes of EUS‐TA

The sensitivity, specificity, and accuracy rates of EUS‐TA were 96.8%, 100%, and 96.8%, respectively, in the ≤2 cm group and 97.4%, 100%, and 97.4%, respectively, in the >2cm group, with no significant differences between the two size groups. Moreover, there was no difference in adverse events between the two groups. There were two cases (2.3%) of mild abdominal pain in the >2 cm group, but the pain resolved spontaneously (Table 3). There were no cases of bleeding, perforation, or infection.

**TABLE 3:** Outcomes of endoscopic ultrasound‐guided tissue acquisitions for focal liver lesions.

	Focal liver lesions ≤2 cm (*n* = 32)	Focal liver lesions >2 cm (*n* = 77)	*p‐*value
Sensitivity	96.5% (22/22)	97.2% (78/81)	0.8
Specificity	N/A (0/0)	100% (6/6)	0.71
Accuracy	100% (22/22)	97.4% (84/87)	0.79
Adverse events, *n* (%)	1 (4.5)	1 (1.1)	0.49

## DISCUSSION

In a recent review, EUS‐TA for solid pancreatic lesions had favorable outcomes, with high diagnostic accuracy and a low incidence of adverse events.^2^, ^7^, ^8^ Although EUS‐TA is an established procedure, the accuracy rate has been reported to decrease in cases of small pancreatic lesions.^3^

Sugiura et al. investigated the effect of pancreatic tumor size on the accuracy rate of EUS‐TA.^9^ The authors analyzed 788 solid pancreatic lesions in 761 patients. All lesions were stratified into five groups based on size as follows: groups A (<10 mm; *n* = 36), B (10–20 mm; *n* = 223), C (20–30 mm; *n* = 304), D (30–40 mm; *n* = 147), and E (≥40 mm; *n* = 78). In groups A, B, C, D, and E, the sensitivities were 89.3%, 95.0%, 97.4%, 98.5%, and 98.7%, respectively, showing a significant increase with an increase in size (*p* < 0.01), and the diagnostic accuracies were 91.7%, 96.4%, 97.7%, 98.6%, and 98.7%, respectively, showing a significant increase with an increase in size (*p* = 0.03). The adverse event rates were not significantly different among the five groups.

Nakai et al. performed a meta‐analysis of 33 studies with 6883 cases.^3^ The authors found that the pooled odds ratio (OR) of sensitivity was significantly higher in solid pancreatic lesions of >20 mm (OR 1.64, *p* = 0.02) and in lesions of >10 mm (OR 3.05, *p* = 0.01), but not in lesions of >30 mm (OR 1.18, *p* = 0.46). The meta‐analysis of accuracy also showed a similar trend: OR of 1.59 in solid pancreatic lesions of >20 mm (*p* < 0.01) and OR of 3.27 in lesions of >10 mm (*p* < 0.01) and OR of 1.03 in lesions of >30 mm (*p* = 0.87). The use of a 25‐gauge needle tended to improve sensitivity in cases of small solid pancreatic lesions; however, this improvement was not statistically significant.

EUS‐TA is technically difficult for small lesions. Even if the puncture is successful, the needle stroke is limited, and thus, the obtained specimen is likely to be small. The inability to obtain an adequate specimen may be responsible for its reduced diagnostic ability in these cases.

Conventionally, ultrasonography‐guided percutaneous biopsy has been performed for focal liver lesions. Percutaneous biopsy for focal liver lesions is an established technique; its accuracy rate is 93.4%–95.1%, with an adverse event rate of 0.1%–10.9%.^10^, ^11^

Nguyen et al. in 1999 first reported the use of EUS‐TA for focal liver lesions.^12^ This approach has been reported to have a favorable diagnostic ability (88%–100%) and a low adverse event rate (0%–6%).^13^, ^14^, ^15^, ^16^, ^17^, ^18^, ^19^, ^20^, ^21^, ^22^, ^23^, ^24^ A study comparing the outcomes of percutaneous biopsy and EUS‐TA for focal liver lesions showed no difference in the diagnostic accuracy rate; however, it reported that EUS‐TA had significantly fewer adverse events, particularly postprocedural pain.^24^

Currently, in Japan, most facilities employ percutaneous biopsy as the first choice for focal liver lesions. However, owing to its high accuracy rate and low adverse event rate, EUS‐TA may become the first‐choice technique to replace percutaneous biopsy. It has been reported that EUS‐TA can be performed even for right lobe lesions.^19^ The greatest advantage of EUS‐TA is that it enables puncture via the digestive tract. For this reason, it is useful for cases that are difficult to handle using the percutaneous approach, including Chilaiditi's syndrome, severe obesity, caudate lobe lesions, and cases with ascites.^24^ Previous studies of EUS‐TA for focal liver lesions have included target lesions having a median size of 11–34 mm, but no studies have focused on the outcomes in cases of small focal liver lesions measuring ≤2 cm.

In this study, we compared the outcomes of EUS‐TA between focal liver lesions measuring ≤2 cm and those measuring >2 cm. There were significantly fewer right lobe lesions and transduodenal punctures in the ≤2 cm group than in the >2 cm group. Case selection may have been influenced by operator bias. Generally, transduodenal puncture is more difficult than transgastric puncture, especially for small focal liver lesions. As EUS‐TA for right lobe lesions measuring ≤2 cm is expected to be technically difficult, other approaches, such as short‐term follow‐up with an imaging modality or percutaneous biopsy, may have been prioritized. In addition, the final pathological diagnosis showed that metastatic liver tumors were more common in the ≤2 cm group, while intrahepatic cholangiocellular carcinoma and gallbladder cancer were more common in the >2 cm group. After the diagnosis and resection of a primary malignant neoplasm, regular whole‐body imaging is performed. Metastatic lesions can often be detected when they are still small. Thus, the ≤2 cm group may have included many metastatic liver tumors that were discovered early.

The accuracy rate and adverse event rate showed no significant differences between the two size groups. Contrary to the initial hypothesis, EUS‐TA for small focal liver lesions had favorable outcomes. Several factors may explain these favorable outcomes. The first factor is the difference in the pathological characteristics of the tumors. Most solid pancreatic lesions for which EUS‐TA is indicated are invasive ductal carcinomas. Pancreatic cancer is pathologically characterized by dense fibrosis.^25^ Thus, even when the target lesion is punctured in EUS‐TA, some cases show only fibrosis and inflammation on pathological examination, without the presence of cancer cells. However, the focal liver lesions in this study (metastatic liver tumors, intrahepatic cholangiocarcinoma, hepatocellular carcinoma, and gallbladder cancer) were rarely accompanied by dense fibrosis, and it might have been easier to collect the tumor cells by needle biopsy.

The second factor is the possibility of greater technical ease of EUS‐TA for focal liver lesions than for pancreatic lesions. EUS‐TA for pancreatic lesions must be performed carefully to not puncture the main pancreatic duct to avoid pancreatic juice leakage and pancreatitis. In EUS‐TA for focal liver lesions, it is necessary to avoid the bile duct and blood vessels (portal vein and artery). However, it is easier to perform EUS‐TA for focal liver lesions than for pancreatic lesions, especially when the target lesion is in the left lobe or caudate lobe and transgastric puncture is performed. In this study, most (87.5%) patients in the ≤2 cm group were punctured via the gastric route, which may have contributed to the favorable outcomes in this group.

The third factor is the definition of small lesions as 2 cm or less. Although the accuracy of EUS‐TA for solid pancreatic lesions ≤2 cm is significantly decreased, this tendency is more pronounced for solid pancreatic lesions ≤1 cm. In the present study, there were only two cases of focal liver lesions ≤1 cm and the majority of lesions of the ≤2 cm group were 10–20 mm in size. If the study had been limited to focal liver lesions ≤1 cm, there may have been a significant difference in the accuracy rate.

In this study, FNA needles were frequently used (70.6%, 77/109). In our institution, FNA needles were used from 2016 to 2020, whereas fine‐needle biopsy needles have been mainly used since 2021. As several cases before 2020 were included, the proportion of FNA needles was high in this study.

In this study, ROSE was not performed. At our institution, performing ROSE in daily clinical practice is difficult owing to a shortage of pathology departments. If ROSE could be performed, evaluating whether appropriate samples were obtained even from small lesions would be possible, which may contribute to reducing the number of punctures.^26^ The following were the limitations of this study: (1) a single‐center retrospective study and the number of cases was relatively small, and (2) bias in case selection cannot be denied. In the future, it would be desirable to conduct clinical studies at multiple institutions with a large number of cases.

EUS‐TA for small focal liver lesions measuring ≤2 cm has favorable outcomes, which are similar to those for lesions measuring >2 cm. EUS‐TA is a useful biopsy method even in cases with small focal liver lesions.

## CONFLICT OF INTEREST STATEMENT

None.

## ETHICS STATEMENT

This is a single‐center, retrospective, observational study that was approved by the ethics committee of Showa University (Approval number: 2023‐195‐B).

## PATIENT CONSENT STATEMENT

N/A
