# Correction to “The outcomes of endoscopic ultrasound‐guided tissue acquisition for small focal liver lesions measuring ≤2 cm”

**DOI:** 10.1002/deo2.70039

**Published:** 2024-11-19

Takano Y, Tamai N, Yamawaki M *et al.* The outcomes of endoscopic ultrasound‐guided tissue acquisition for small focal liver lesions measuring ≤2 cm. *DEN Open* 2025; **5**: e70031.

1. In the abstract section, the next “The sensitivity, specificity, and accuracy rates were 96.8%, 100%, and 96.8%, respectively, in the ≤2 cm group and 97.4%, 100%, and 97.4%, respectively, in the >2 cm group, with no significant differences between the groups. There was no difference in adverse events between the groups (0% in the ≤2 cm group and 2.3% in the >2 cm group).” was incorrect.

This should have read: “The sensitivity, specificity, and accuracy rates were 96.5%, 100%, and 96.8%, respectively, in the ≤2 cm group and 97.2%, 100%, and 97.4%, respectively, in the >2 cm group, with no significant differences between the groups. There was no difference in adverse events between the groups (0% in the ≤2 cm group and 2.5% in the >2 cm group). ” (Please correct the underlined numbers.)

2. In the result outcomes of the EUS‐TA section, the next “The sensitivity, specificity, and accuracy rates of EUS‐TA were 96.8%, 100%, and 96.8%, respectively, in the ≤2 cm group and 97.4%, 100%, and 97.4%, respectively, in the >2cm group, with no significant differences between the two size groups. Moreover, there was no difference in adverse events between the two groups. There were two cases (2.3%) of mild abdominal pain in the >2 cm group, but the pain resolved spontaneously.” was incorrect

This should have read: “The sensitivity, specificity, and accuracy rates of EUS‐TA were 96.5%, 100%, and 96.8%, respectively, in the ≤2 cm group and 97.2%, 100%, and 97.4%, respectively, in the >2 cm group, with no significant differences between the two size groups. Moreover, there was no difference in adverse events between the two groups. There were two cases (2.5%) of mild abdominal pain in the >2 cm group, but the pain resolved spontaneously.” (Please correct the underlined numbers.)

3. The numbers in Table 3 are incorrect.

**Table:** 

	Focal liver lesions ≤2 cm (*N* = 32)	Focal liver lesions >2 cm (*N* = 77)	*p*‐Value
Sensitivity	96.5% (22/22)	97.2% (78/81)	0.8
Specificity	N/A (0/0)	100% (6/6)	0.71
Accuracy	100% (22/22)	97.4% (84/87)	0.79
Adverse events, no. (%)	1 (4.5)	1 (1.1)	0.49

The correct table is listed below: Please correct the underlined numbers.

**Table:** 

	Focal liver lesions ≤2 cm (*N* = 32)	Focal liver lesions >2 cm (*N* = 77)	*p*‐Value
Sensitivity	96.5% (28/29)	97.2% (72/74)	0.8
Specificity	100% (3/3)	100% (3/3)	0.71
Accuracy	96.8% (31/32)	97.4% (75/77)	0.79
Adverse events, no. (%)	0 (0)	2 (2.5)	0.49

We apologize for this error.

